# Knowledge of Problem Solving (KOPS) Scale: Design and Evaluation of a Digitally Administered Competence Measure for a Common Practice Element in Task-Shared Youth Mental Health Interventions

**DOI:** 10.1007/s41347-023-00356-9

**Published:** 2023-10-21

**Authors:** Sonal Mathur, Daniel Michelson, Tejaswi Shetty, Vikram Patel, Andy P. Field

**Affiliations:** 1 Sangath, New Delhi, India; 2 Department of Child & Adolescent Psychiatry, Institute of Psychiatry, Psychology & Neuroscience, King’s College London, London, UK; 3 Department of Global Health and Social Medicine, Harvard Medical School, Boston, USA; 4 Harvard T.H. Chan School of Public Health, Boston, USA; 5 School of Psychology, University of Sussex, Brighton, UK

**Keywords:** Problem-solving intervention, Adolescent mental health, Digital technology, Psychometrics, Measure development, Capacity building, India

## Abstract

There are few reliable and feasible quality assurance methods to support scaling up of psychological interventions delivered by non-specialist providers. This paper reports on the phased development and validation of a digitally administered Knowledge of Problem Solving (KOPS) measure to assess competencies associated with a “task-shared” problem-solving intervention for adolescents with diverse mental health problems in India. Phase 1 established key competencies required to deliver the intervention, followed by item generation for a corresponding knowledge-based competency measure that could be administered efficiently through e-learning systems. In phase 2, items were refined based on responses from an “experienced” reference sample comprising 17 existing counsellors with direct experience of the problem-solving intervention, and a “novice” sample with 14 untrained university students and NGO staff. In phase 3, we evaluated two parallel versions of the measure in a validation sample (*N* = 277) drawn from universities and NGOs. The resulting 17-item measure was structured around a hypothetical case, followed by multiple-choice questions that asked about the most appropriate response to a practice-based scenario. The difficulty level of the test items was well matched to the ability level of participants (i.e. most items being of moderate difficulty and few items being easy or difficult). Only one item showed a negative discrimination index and was removed from the 17-item forms. The final 16-item version of the KOPS measure provides a scalable digital method to assess key psychotherapeutic competencies among non-specialists, particularly in relation to a transdiagnostic problem-solving intervention. Similar formats could be deployed more widely alongside e-learning programmes to expand the global workforce capable of delivering evidence-based psychological interventions.

**Supplementary Information:**

The online version contains supplementary material available at 10.1007/s41347-023-00356-9.

## Introduction

Task-sharing is an established strategy for building mental health service capacity, especially in low- and middle-income countries. Task-sharing often involves the training of non-specialist providers (NSPs) to deliver evidence-based psychological interventions (Hoeft et al., 2018; Scott et al., 2018;. A major barrier to scaling up task-sharing approaches is the reliance on traditional models of in-person, expert-led training workshops for NSPs (Philippe et al., 2022; van Ginneken et al., 2021). Attention has therefore focused on less-resource intensive training models, and particularly those involving digital technologies that enable efficiencies in training resources and in post-training support (Naslund et al., 2019; O’Connor et al., 2018).

A promising approach to task-sharing in the area of adolescent mental health has been developed by Sangath NGO and international collaborators as part of the Premium for Adolescents (PRIDE) programme in India. PRIDE was initiated in 2016 and concluded in 2022, with the overall aim to establish a transdiagnostic, stepped care model addressing common mental health problems in Indian secondary schools (Michelson et al., 2020a). A first-line problem-solving intervention (“Step 1”) was designed in a brief (3-week) face-to-face format utilising “lay” counsellors, and tested against problem-solving booklets alone in a large randomised controlled trial (Michelson et al., 2020b). The counselling format had significant effects on self-reported psychosocial problem severity at 6 and 12 weeks, as well as sustained effects on psychosocial problems and mental health symptoms over 12 months (Malik et al., 2021).

Subsequent efforts to scale up the Step 1 intervention in India have involved updating an existing e-learning platform, which was originally set up to train prospective providers in a brief intervention for adults with depression (Muke et al., 2020). The platform can be accessed through any internet-enabled device, for example a smartphone or a computer, and content can be followed independently (i.e. as a self-guided programme) or with external coaching. A randomised controlled trial has investigated the relative effects of self-directed and coach-supported training for prospective Step 1 providers, demonstrating a significant incremental benefit of coaching on knowledge-based competencies (Mathur et al., 2023a, b).

In this brief report, we describe the design and preliminary psychometric evaluation of a competency assessment measure that was chosen as the primary outcome in the aforementioned PRIDE training trial (see Mathur et al., 2023a) for complete trial protocol). The Knowledge of Problem Solving (KOPS) scale incorporates a vignette-based, multiple-choice question–answer format. Its design is intended to address key feasibility challenges in scaling up expert-rated observational scales (Cooper et al., 2015; Kohrt et al., 2015; Ottman et al., 2020). The user-friendly digital format permits efficient self-administration, while the vignettes reflect diverse therapeutic scenarios that may be encountered in real-world settings. Although initially intended for use in India, there may also be wider applications for the measure given that problem-solving is among the most common practice elements in evidence-based psychological interventions for adolescents worldwide (Michelson et al., 2022).

## Methods

### Design

We followed a phased approach in line with previous research on scalable psychotherapeutic competency measures (Cooper et al., 2015; Restivo et al., 2020). Phase 1 developed a competency “blueprint” that outlined the knowledge and applied skills required to deliver a transdiagnostic problem-solving intervention. Phase 2 involved drafting two parallel versions of the competency measure. Phase 3 tested the psychometric properties of these parallel forms.

### Participants

Following on from desk-based activities in phase 1 (see procedures below), phase 2 involved 14 individuals without experience of providing mental health services of any type (the “novice” group), and 17 individuals who had already been trained in the PRIDE problem-solving intervention (the “experienced” group). The novice group comprised university students studying psychology, education, or allied disciplines and NGO staff working with adolescents. The experienced group was recruited from among Sangath staff who were not otherwise directly involved in designing the KOPS scale.

Phase 3 was embedded within a larger study that used a randomised controlled trial design to evaluate two digital formats (with and without coaching) for training prospective NSPs in the PRIDE problem-solving intervention (Mathur et al., 2023a). A total of *N* = 277 trial participants were recruited from four universities (located in Delhi, Bangalore, and Mumbai) and five NGOs working in the fields of adolescent health and education. The current study uses data collected from trial participants at baseline (i.e. before allocation to a training condition).

### Phase 1: Selection of Competencies

#### Procedures and Interim Findings

A working group comprising 3 India-based authors (SM, RM, TS) reviewed the existing PRIDE intervention manual and training materials (Michelson et al., 2020a, b) to generate lists of non-specific counselling competencies (e.g. rapport building; verbal and non-verbal communication) and competencies that are specific to problem-solving (e.g. identifying problems; selecting and implementing solutions). An initial blueprint was reviewed by six experts comprising original developers of the problem-solving intervention and other clinical experts, as well as a separate group comprising eight NSPs who had been previously trained to deliver the intervention in question. These reviewers were independent from the working group and advised on the extent to which the blueprint achieved adequate coverage of key competencies needed for effective delivery of a transdiagnostic problem-solving intervention (Table 1). Experts and NSPs rated individual items (from 1 = lowest to 3 = highest) according to their relative importance, and provided additional qualitative feedback on the distinctiveness and redundancy of respective competencies. To enhance external validity, competency domains were also cross-referenced with problem-solving competencies from a widely cited CBT competency framework that has been used in large-scale training of psychological practitioners elsewhere (Roth & Pilling, 2008). Based on feedback and external comparisons, items were consolidated, removed, or added. The final blueprint (see Supplementary Materials, Table I-SM) covered 18 competencies, 13 of which were non-specific and five were specific to problem-solving.

**Table 1:** Ratings of relative importance for candidate competencies (phase 1 blueprint)

Competency	Expert ratings (mean)	Non-specialist ratings (mean)
Working collaboratively	2.83	2.75
Demonstrating empathy, warmth, and genuineness	2.83	3
Non-judgmental attitude	2.83	2.75
Active listening	2.5	3
Summarizing	2.33	2.63
Hope building, positive feedback and encouragement	2.5	2.88
Asking questions	2.66	3
Using appropriate language	2.5	2.63
Building rapport	2.33	2.85
Shared decision-making	3	2.88
Assessing and ensuring progress	2.5	2.75
Home practice	2.83	2.75
Assessing and managing self-harm	3	3
Addresses barriers	2.5	2.63
Explaining confidentiality	2.83	2.88
Documentation	3	2.63
Structuring sessions	2.67	2.75
External referrals	2.2	2.63
Understanding mental health problems in adolescents	2.83	2.75
Starting problem-solving	2.66	2.88
Identifying target problem	3	2.75
Generating options	3	2.88
Developing action plan for problem-solving	3	2.88
Reviewing action plan	2.83	2.88
Consolidating learning and ending counselling	2.83	3

Ratings ranged from 1 to 3, where higher scores indicate higher relevance

### Phase 2: Item Generation

#### Procedures and Interim Findings

Item generation was guided by established principles for creating multiple-choice quizzes (MCQs) (Haladyna, 2004; Plake & Wise, 2014). The objective was to create two parallel MCQ forms to permit repeat assessment without practice effects. Two independent teams each created a unique case description reflecting common adolescent mental health problems in the study setting, as well as generating a series of plausible counselling vignettes that followed from the case description. Similar formats have been used for assessing competencies of NSPs in other low-resource settings, though not in relation to youth-focused psychosocial interventions or problem-solving specifically (Asher et al., 2021; Ottman et al., 2020).

Each form began with a briefing note about the case’s presenting problems and context. This was followed by five vignettes, each pertaining to a different counselling session with the same case, arranged in sequential order. Each of the vignettes was accompanied by either 3 or 4 questions, making a total of 18 questions and with each question intended to assess a different competency. All items were designed as one-best-answer multiple-choice questions, which consisted of a lead-in question followed by four answer options. Items focused on assessing applied knowledge (i.e. knowing how to implement the intervention in a given situation) rather than theoretical knowledge, as recommended in other pedagogical research (Carneson et al., 1996). To create plausible but incorrect “distractors”, we referred to a list of common errors/misconceptions that the PRIDE supervisors had noted over the course of 5 years spent training and supervising NSPs (e.g. the misconception that confidentiality must never be broken under any circumstance).

Two parallel versions of the measure, each comprising 18 items, were subsequently piloted. Items that were correctly answered by more than 35% of the novice group were deemed to be too easy and vulnerable to guessing. Conversely, items for which less than 65% of the experienced group could answer correctly were deemed to be too difficult/ambiguous. Cognitive interviews were additionally conducted with two novices and two experienced individuals, which helped to ensure that the items were clearly worded and had one best answer. Out of 36 items (18 in each version), 19 were refined, mainly by re-wording the incorrect options or distractors in such a way that would improve their discrimination ability. Fifteen items were removed due to their ambiguity and replaced with newly drafted items. Two items which corresponded separately to “risk assessment” and “risk management” were combined. Thus, two parallel 17-item measures were generated, with each competency represented by a single question.

### Phase 3: Psychometric Evaluation

#### Procedures

The study was hosted on the REDCap platform, which permits creation, administration, and management of online surveys (Harris et al., 2019). After providing demographic information and informed consent, participants were randomised to receive one of the two parallel forms. The randomisation sequence was programmed into REDCap. Upon randomisation, participants were automatically presented with the relevant KOPS form. The forms were available in both English and Hindi and participants could choose their preferred language. Each participant was provided with 90 min to complete the measure.

#### Data Analysis

Rasch analysis is a commonly used psychometric method for developing assessment tools in educational contexts. It is based on item-response theory (Fischer & Molenaar, 2012) and involves estimating an item characteristic curve (ICC) for each item showing the probability of a correct response as a function of the respondent’s ability/knowledge. Ideally, the curve is S-shaped meaning a low probability of a correct response when ability is also low, and an increasing probability of a correct response as ability increases. The horizontal position of the curve is the *difficulty parameter*: curves centred to the left of “average ability” represent easy items (the probability of a correct response is high even for those with low ability); curves centred to the right of “average ability” represent difficult items (the probability of a correct response is low even for those with high ability). The slope of the curve is the *discrimination parameter*: flat curves suggest that an item cannot discriminate between respondents with different ability; steep curves discriminate well. The analysis also yields a test information curve (TIC) indicating the information provided by the test (*y*) as a function of ability (*x*). Ideally this curve is flat, indicating that the information provided by the test is equal for all ability levels. In reality, the curve is commonly bell-shaped indicating that the least information is provided for people with extremely low or high ability.

The aim of our analysis was to identify items that were psychometrically weak, and to evaluate the information characteristics of the test overall. That is, to identify items that may be too difficult or easy (based on the difficulty parameter), or discriminate poorly (based on the discrimination parameter) while also considering the overall shape of the test information curve. Analyses were carried out using R 4.2 (R Core Team, 2002) and the ltm (Rizopoulos, 2006) and tidyverse (Wickham et al., 2019) R packages.

### Findings: Test Completion

In total, *N* = 277 individuals completed one of the two versions of the KOPS competency measure (*n* = 123 for Form A; *n* = 154 for Form B). This imbalance was because the original randomisation sequence was generated for 500 participants. The mean age of participants was 26.1 years (SD = 7.1). Most participants were female (*n* = 229, 82.7%) and included a mix of university students (*n* = 122, 44%) and NGO workers (*n* = 155, 56%). In terms of the participants’ highest level of completed education, *n* = 126 (45.5%) held a bachelor’s degree; *n* = 86 (31.0%) held a post-high school diploma or equivalent; *n* = 63 (22.7%) held a master’s degree; and *n* = 2 (0.7%) had completed education up to 12^th^ standard (i.e. had finished high school).

### Findings: Rasch Analysis

**Fig. 1:**
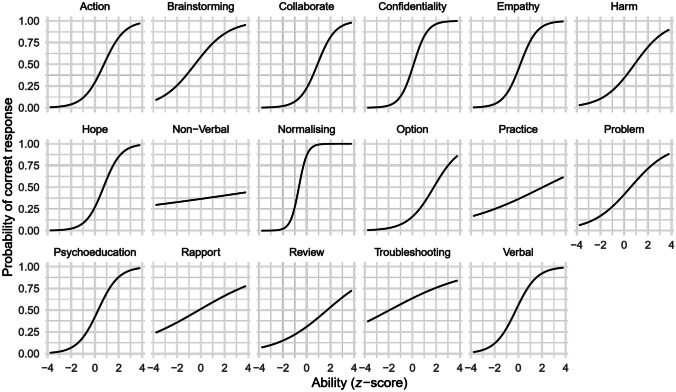
Item characteristics curve for Form A

**Fig. 2:**
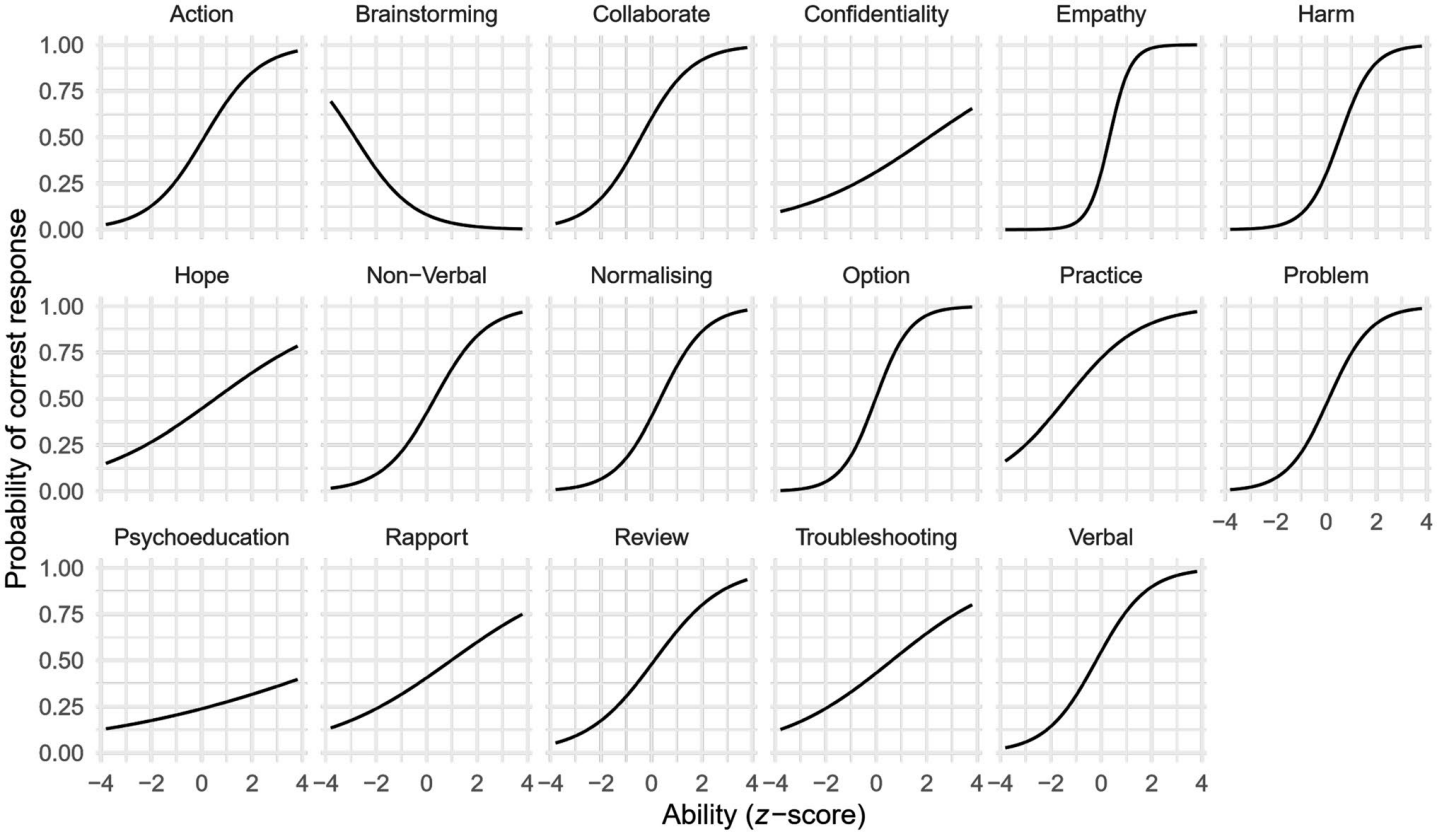
Item characteristics curve for Form B

Forms A and B were fairly well matched in terms of overall item difficulty (see Table 2), discrimination (see Table 3), and the overall TICs (see Figs. 1 and 2). Thus, the forms appeared to be similar in terms of the information they provide at different ability levels. Figures 1 and 2 also show that although the most information was provided about individuals of average ability, the curves had a reasonable spread around the average. This pattern suggests that the forms provided some information about individuals above and below average ability, but not so much at the extremes.

**Table 2:** Number of items falling into each difficulty category

**Form**	**Very easy**	**Easy**	**Moderately difficult**	**Difficult**	**Very difficult**
A	0	1	12	2	2
B	1	1	13	0	2

**Table 3:** Number of items falling into each discrimination category

**Form**	**Poor**	**Low**	**Moderate**	**High**	**Very high**
A	4	2	7	2	2
B	2	4	8	2	1

In terms of the ICC curves, the difficulty columns in Tables 4 and 5 show that most items were moderately difficult for both forms, with a few very easy items (one on Form A, two on Form B), and a few very difficult items (two on each form). This mix was reasonable. The discrimination columns in Tables 4 and 5 show that, on both forms, 11 items had moderate to high discrimination (above 0.65), and 4 items (Form A) and 2 items (Form B) had potentially problematic low discrimination (below 0.35). The column *P (correct|average)* in Tables 4 and 5 indicates the probability of a correct response for respondents of average ability. Most items (12 for Form A, 13 for Form B) had a probability below 0.5, suggesting that a respondent of average ability would choose an incorrect response more often than a correct one.

**Table 4:** Item analysis of Form A

**Competency**	**Difficulty**	**Discrimination**	***P (correct|average)***
Exploring and normalising feelings (Normalising)	−0.65	2.84	0.86
Troubleshooting (Troubleshooting)	−1.93	0.29	0.64
Generating options (Option)	−0.49	0.70	0.59
Verbal communication (Verbal)	−0.20	1.13	0.56
Building rapport (Rapport)	−0.16	0.32	0.51
Explaining confidentiality (Confidentiality)	0.10	1.72	0.46
Demonstrating empathy, warmth, and genuineness (Empathy)	0.17	1.43	0.44
Psychoeducation with local terminology (Psychoeducation)	0.27	1.15	0.42
Identifying target problem (Problem)	0.54	0.62	0.42
Non-verbal communication (Non-verbal)	6.676	0.08	0.37
Home practice (Practice)	2.06	0.27	0.36
Assessing and managing self-harm (Harm)	0.88	0.75	0.34
Reviewing action plan (Review)	1.72	0.47	0.31
Developing action plan for problem-solving (Action)	0.76	1.16	0.29
Promoting realistic hope for change (Hope)	0.72	1.36	0.27
Working collaboratively (Collaborate)	0.92	1.34	0.23
Selecting option(s) (Option)	1.80	0.92	0.16

Short-hand labels in parentheses correspond to graph headings used in Figs. 1 and 2

**Table 5:** Item analysis of Form B

**Competency**	**Difficulty**	**Discrimination**	***P (correct|average)***
Home practice (Practice)	−1.39	0.68	0.72
Working collaboratively (Collaborate)	−0.41	1.00	0.60
Verbal communication (Verbal)	−0.19	0.99	0.55
Selecting option(s) (Option)	−0.02	1.46	0.51
Developing action plan for problem-solving (Action)	0.10	0.91	0.48
Reviewing action plan (Review)	0.12	0.73	0.48
Identifying target problem (Problem)	0.11	1.21	0.47
Promoting realistic hope for change (Hope)	0.54	0.40	0.45
Troubleshooting	0.62	0.44	0.43
Non-verbal communication (Non-verbal)	0.32	0.98	0.42
Building rapport (Rapport)	0.98	0.39	0.41
Exploring and normalising feelings (Normalising)	0.35	1.12	0.40
Demonstrating empathy, warmth, and genuineness (Empathy)	0.33	2.41	0.31
Explaining confidentiality (Confidentiality)	2.09	0.38	0.31
Assessing and managing self-harm (Harm)	0.55	1.54	0.30
Psychoeducation with local terminology (Psychoeducation)	5.93	0.20	0.24
Generating options (Option)	−2.84	−0.85	0.08

Short-hand labels in parentheses correspond to graph headings used in Figs. 1 and 2

Given the positive characteristics of the TIC and the generally good range of discrimination and difficulty across items, most items were deemed useful. The exception was the item for “brainstorming” on Form B, which had a negative discrimination index and extremely low difficulty. Although removing this item had almost no impact on the TIC, it was rejected along with its counterpart on Form A, thus keeping the forms balanced. The final measure therefore consisted of 16 items, each linked to a unique competency as listed in Table 6, and available in parallel forms.

**Table 6:** Final list of 16 assessed competencies

**Competency**
**Non-specific competencies**
Non-verbal communication
Verbal communication
Explaining confidentiality
Building rapport
Exploring and normalising feelings
Demonstrating empathy, warmth, and genuineness
Assessing and managing self-harm
Working collaboratively
Promoting realistic hope for change
Psychoeducation with local terminology
Home practice
Troubleshooting
**Problem-solving competencies**
Identifying target problem
Selecting option(s)
Developing action plan for problem-solving
Reviewing action plan

## Discussion

This study developed and validated the KOPS measure: a brief, scalable measure that can be used to assess the competency of non-specialists to deliver a problem-solving intervention for adolescents with common mental health problems. Two versions of the measure with equivalent difficulty levels were developed to allow repeated testing of training outcomes over time without practice effects. The difficulty of the test items was well matched to the ability level of trainees, with most items being of moderate difficulty and few items being easy or difficult, which is ideal for a test (Case & Swanson, 1998).

Competency measures are vital for ensuring that non-specialists have acquired the key knowledge and skills needed to undertake new mental health care roles. Typically, these measures have been designed for use with structured observations of actual sessions or analogue situations with “clients” (Fairburn & Cooper, 2011). Such observational formats require skilled assessors who can reliably identify and rate practices, but who are typically in short supply in many global settings. In addition to being labour-intensive, there are also practical challenges of in vivo and role-play assessments, such as role-played situations feeling inauthentic (particularly when an adult is playing the role of a child or adolescent); trainees’ anxiety at being observed and rated; and the inherent variability of real-life cases (Cooper et al., 2017; El Masri et al., 2023). Observational assessments are especially impractical when trainees are accessing training and supervision remotely via e-learning platforms. Although recorded sessions can potentially be uploaded to file servers or emailed, this is harder in low-resource settings with weak digital infrastructure. Distance and technical barriers can also undermine the authenticity of role-plays conducted online (Young, 2022).

The KOPS measure obviates the need for skilled assessors and can be self-administered and scored in a relatively simple digital format. The use of written case vignettes, designed with input from local practitioners, enables respondents to apply their knowledge to practice-based scenarios that reflect common presenting problems and process issues encountered in the field. Questions are arranged in sequential order following the chronology of a multi-session intervention. These characteristics offer further advantages, in terms of external validity, relative to knowledge-based quiz formats that emphasise theoretical aspects of psychotherapy over practical applications of knowledge (Myles & Milne, 2004).

This study has several strengths. In developing our measure of provider competency, a rigorous stepwise approach was used to achieve two test forms with equivalent difficulty levels. We followed an iterative process in developing our KOPS competency blueprint, triangulating content from an existing problem-solving intervention manual and associated training curriculum; an independent competency framework that included problem-solving and non-specific competencies; and incorporating formative feedback from experienced clinicians as well as novices. Moreover, items were pre-tested on a relatively large sample of novice practitioners including university students and NGO workers who closely resembled the intended training population.

We acknowledge that the measure does not directly assess the applicability of the acquired knowledge in real-life settings and we did not obtain data on its predictive validity with respect to clinical outcomes. These would be future research directions along with concurrent validation against the “gold-standard” of role-plays to evaluate the concordance of knowledge-based competency with skill-based assessment.

## Conclusions

Competency measures are an integral part of quality assurance in psychological therapies. Previous research has recognised that many existing competency measures involve observer-rated formats that are prohibitively expensive and time consuming for routine use, highlighting the need to strike a balance between reliability, validity, and feasibility (Muse & McManus, 2013; Ottman et al., 2019). The digitally administered approach used in the current study could be rapidly extended to other contexts, offering a scalable methodology for developing and evaluating knowledge-based competencies for NSPs engaged in task-sharing of psychological interventions across a range of settings.

### Supplementary Information

Below is the link to the electronic supplementary material.

Supplementary file1 (DOCX 34.5 KB)

## Data Availability

Data may be obtained from the principal investigator (Vikram Patel) subject to reasonable request.
