# Supplementary material for: Knowledge of Problem Solving (KOPS) Scale: Design and Evaluation of a Digitally Administered Competence Measure for a Common Practice Element in Task-Shared Youth Mental Health Interventions
Source: J Technol Behav Sci. 2023 Oct 21;9(3):418–27. doi: 10.1007/s41347-023-00356-9 (PMC11330393; doi:10.1007/s41347-023-00356-9)
Supplement: Supplementary file 1 — Supplementary file1 (DOCX 34.5 KB) [file 41347_2023_356_MOESM1_ESM.docx]

**Table I-SM. Original list of 18 assessed competencies**

| **Number** | **Competency** |
| --- | --- |
| **Non-specific competencies** | |
| 1 | Non-verbal communication |
| 2 | Verbal communication |
| 3 | Explaining confidentiality |
| 4 | Building rapport |
| 5 | Exploring & normalising feelings |
| 6 | Demonstrating empathy, warmth & genuineness |
| 7 | Assessing self-harm |
| 8 | Managing self-harm |
| 9 | Working collaboratively |
| 10 | Promoting realistic hope for change |
| 11 | Psychoeducation with local terminology |
| 12 | Home practice |
| 13 | Troubleshooting |
| **Problem-solving competencies** | |
| 14 | Identifying target problem |
| 15 | Brainstorming options |
| 16 | Selecting option(s) |
| 17 | Developing action plan for problem solving |
| 18 | Reviewing action plan |

**Final KOPS measure (16-item versions)**

**Why do I have to complete this assessment?**

The purpose of this assessment is to measure your knowledge of general counselling and specific problem-solving skills. Your responses will help us to understand the effect of the course on participants’ knowledge.

**What do I need to do?**

We request that you complete the assessment in a quiet place with good internet connectivity. Please ensure that you are by yourself and do not share these questions with anyone else. We also urge you to complete the assessment in one sitting and NOT to consult external sources. Your performance will not be shared with anybody else. It will not affect your eligibility to take part in the course.

**How much time do I have?**

You will have **90 minutes** to complete the assessment. After this, the link to the assessment will expire and you will not be able to access the assessment.

**What does the assessment involve?**

- You will be presented with a series of hypothetical counselling scenarios related to a particular adolescent case.
- The counselling scenarios are organised into 5 parts and will be presented in sequential order.
- You will have to read each scenario and answer the related multiple-choice questions (MCQs). The MCQs will ask about appropriate responses to the hypothetical scenario.
- There are a total of 16 MCQs for you to answer.
- Each MCQ has four response options for you to choose from. You will be able to choose ONE answer – the one you think is the most suitable. If you are unsure what answer to choose, please make your best guess.
- Each correct answer will be awarded ‘1’ point and each incorrect answer will receive ‘0’ points.

**Scenario A**

*Lakshmi is a 15-year-old girl studying in the 10th Standard. She lives with both her parents, and two younger siblings who are 13 and 10 years old. This is an important year for her, as she is preparing for her class X board exams. However, she is finding it difficult to concentrate on her studies. She has recently performed poorly in the pre-board exams, where she could not score the passing marks for two subjects, Maths and Science. She usually enjoys participating in extracurricular sports activities, but her parents have not allowed her to play sports this year so that she can focus on academics. Her younger sisters have been doing very well in their studies and one of them is consistently obtaining 1st rank in her class. Her parents frequently compare Lakshmi unfavourably with her younger sisters.*

*Lakshmi has one month to prepare for her upcoming board exams. She is feeling unmotivated and lacking in energy. She worries every day about her studies and performance in the upcoming exams. She feels stuck and sometimes thinks of running away so that she does not have to take her exams. She used to be very social and had a large group of friends but now she does not feel like speaking to anyone. She has come for counselling because a friend mentioned that she might feel better if she discusses her problems with a counsellor.*

**Segment 1 instructions**: It is your first session with Lakshmi and you are meeting her for the first time. Read the segment below and answer questions 1-4.

**Lakshmi says the following:**

*“My friend suggested that I should come and see you. She thinks it may help me feel better. I have so many things to do, but I don’t feel like doing anything. I don’t seem to care anymore. I don't even feel like talking to my friends. I feel like running away and sometimes wonder if there is any point in living like this. I don’t know if you can help me or not.”*

Q 1. What might you do to make Lakshmi feel more at ease when meeting for the first time?

1. Introduce yourself and ask more about Lakshmi’s reasons for coming today.
2. Introduce yourself and share your personal experience of similar difficulties.
3. Introduce yourself and explain the potential outcomes of counselling.
4. Introduce yourself and mention your counselling certifications.

Q 2. Lakshmi has mentioned her thoughts of not wanting to live anymore. She further requests that you do not inform her parents about these thoughts. How might you respond?

1. Explain to Lakshmi that since she is a minor, you will have to inform her parents.
2. Explain to Lakshmi that discussions from sessions will remain confidential at all times, and you will not inform her parents.
3. Explain to Lakshmi that if you assess her to be at risk, you will first discuss it with her and then inform her parents.
4. Explain to Lakshmi that as long as she promises you that she won’t harm herself you will not inform her parents.

Q 3. How might you explain a problem-solving counselling approach to Lakshmi?

1. Counselling will help you to vent about your problems.
2. Counselling will help you to bring positive changes in your personality.
3. Counselling will help you to gain insights into the cause of your distress.
4. Counselling will help you to learn ways to manage your problems.

Q 4. Lakshmi has mentioned that she experiences thoughts of ending her life once or twice in a week which last for a few minutes. What would be an appropriate way to manage Lakshmi’s risk of suicide?

1. Encourage Lakshmi to discuss her thoughts of ending her life with her parents.
2. Encourage her to focus on her reasons to continue living.
3. Encourage her to make a contract with you in which she agrees not to harm herself.
4. Encourage her to inform you if she becomes concerned about acting on the thoughts of ending her life.

**Segment 2 instructions**: Your first session with Lakshmi continues. Read the segment below and answer questions 5-8.

**Lakshmi says the following:**

*“My board exams are almost here, and I am failing 2 subjects. It is so difficult for me. I am unable to focus and that makes me very anxious. I usually fall asleep instead of studying, and that makes me feel okay for a while after I wake up. Well...it doesn’t keep the anxious feeling away for very long but it is helpful for that time.*

*My parents keep comparing me to my younger sister who is always topping her class. This makes me feel very useless. Adding to that, now my parents are not letting me participate in any activities, which frustrates me even more! I just wish I could do something better so that my parents are happy with me.*

*There are so many problems in my life I don’t even know where to begin or if anything will help.”*

Q 5. How might you respond following Lakshmi's description of her problems?

1. Summarize Lakshmi's problems and check if you have understood them correctly.
2. Summarize Lakshmi's problems and ask why she is so worried about doing well.
3. Summarize Lakshmi's problems and explore details of how she gets along with her sister.
4. Summarize Lakshmi's problems and then make notes of the conversation.

Q 6. Which of the following statements could be used to validate the difficulties experienced by Lakshmi?

1. “I can see that this is distressing for you. Your situation is unusual since it’s usually the younger siblings who face comparisons.”
2. “I can see that this is distressing for you. It can be stressful to face the pressure of exams and constant comparisons with someone else.”
3. “I can see that this is distressing for you. But don’t worry, in a few years from now your exam marks won’t matter so much.”
4. “I can see that this is distressing for you. I am sorry that your parents are acting like this but I know you understand it is for your own well-being.”

Q 7. You can see that Lakshmi is overwhelmed with her problems and desperate for things to improve. How would you respond to her?

1. Acknowledge her feelings of overwhelm, and explain that things will change now that she is attending counselling.
2. Acknowledge her feelings of overwhelm, and explain that unless she believes things can improve, she won’t put in efforts.
3. Acknowledge her feelings of overwhelm, and explain that you appreciate her coming for counselling despite her apprehensions.
4. Acknowledge her feelings of overwhelm, and explain that there are other ways to view the situation.

Q 8. Lakshmi is confused about what problems to focus on in counselling. What would be your next steps here?

1. Encourage Lakshmi to list the problems and begin to work on them one by one.
2. Encourage Lakshmi to list the problems and identify the one that is most concerning to her.
3. Encourage Lakshmi to list the problems and rank them from easiest to hardest.
4. Encourage Lakshmi to list the problems and choose the problem which has been present for the longest period of time.

**Segment 3 instructions** It is your second session with Lakshmi. You both have decided to work on her difficulty concentrating when she studies. You are facilitating a discussion about options to for solving this problem. Read the segment below and answer questions 9-10.

**Lakshmi says the following:**

*“I’m glad we’re having a session today. This week has been quite bad, with lot of stressful situations that I want to tell you about. I know I had to come up with some options for my problem. I thought that maybe (long pause), maybe I can take my friend’s help to study. If I sit with her to study when she is studying, I will have some company. I can’t be too sure if this is an option for my problem, but I can’t think of anything else. You are the counsellor, you are the expert; can you tell me some options?”*

Q 9. Which of the following tasks would NOT be appropriate for setting the session agenda?

1. Share the points you intend to cover in the agenda.
2. Check with Lakshmi if she would like to add to the agenda.
3. Assess if Lakshmi’s points should be included in the agenda.
4. Ask Lakshmi for her thoughts about the agenda.

Q 10. Lakshmi seems to be feeling hopeless about managing her problems. What would be an appropriate way to make Lakshmi feel hopeful?

1. Assure her that she has adequate preparation time to do well in her exams.
2. Assure her that counselling is a process where the benefits often come with time.
3. Assure her that her parents will appreciate her as much as they appreciate her sister.
4. Assure her that thinking positively will lead to positive results.

**Segment 4 instructions:** It is your third session with Lakshmi. You are helping her to choose options for solving her target problem. Read the segment below and then answer questions 11-13.

**Lakshmi says the following:**

*“I have thought of some options to help me study better but I don’t know if they will work. I have already stopped doing extracurricular activities, but I can also put my mobile phone away or study for 4 hours every night. Another thing I could do is to get our internet disconnected because I get distracted by my mobile or computer when there is internet. Oh, I can even make a timetable to plan what to study. Could you suggest what I should do?”*

Q 11. Lakshmi has identified some options that may help with her target problem. What kind of option would be especially suitable for trying out in an action plan?

1. An option that has relatively few potential disadvantages.
2. An option that requires the least amount of effort from her side.
3. An option that would be enjoyable to try out.
4. An option that works with more than one problem at the same time.

Q 12. Lakshmi is unsure if she can implement the action plan on her own. How might you respond to this doubt?

1. Encourage her to try out the action plan, and advise her not to be afraid of failing because failure is a part of life.
2. Encourage her to try out the action plan and assure her that you are confident it will work.
3. Encourage her to try out the action plan, and ask her to contact you if she faces any challenges.
4. Encourage her to try out the action plan and help identify someone in her life who could assist with it.

Q 13. Lakshmi has to try out the plan as part of her home practice. Which of the following would NOT be an appropriate way to explain the importance of home practice to Lakshmi?

1. Explain that research has shown benefits of home practice in counselling.
2. Explain that doing home practice would prove her commitment to counselling.
3. Explain that you have seen many adolescents benefit from home practice.
4. Explain that she can become expert in what she is learning through home practice.

**Segment 5 instructions:** Lakshmi decides on an action plan that involves following a study timetable. The following segment is from your fourth session with Lakshmi, when you will be reviewing the implementation of the plan. Read the text below and answer questions 14-16.

**Lakshmi says the following:**

*“I was very happy that my plan to follow a timetable actually started working. I was able to concentrate better and I am feeling hopeful that I will be able to do much better than I had earlier thought. Thank you so much, counselling has really helped me learn how to manage my problems. I don’t know what I’ll do without it.”*

Q 14. Which of the following would NOT be a suitable way to review the implementation of Lakshmi’s action plan?

1. Review her intentions behind implementing the plan.
2. Review situations in which she implemented the plan.
3. Review challenges she faced in implementing the plan.
4. Review support she availed in implementing the plan.

Q 15. Lakshmi is silent at one point during the session, following a question that you have asked. It would be appropriate to:

Respond by repeating your question slowly so that Lakshmi is clear about what was asked.

Respond by completing your notes while waiting for Lakshmi’s answer.

Respond by maintaining periodic eye contact while waiting for Lakshmi’s answer.

Respond by telling Lakshmi not to overthink her answer.

Q 16**.** Lakshmi is reluctant to end counselling since she is apprehensive about managing her problems without counsellor support. How would you address this concern?

1. Recognise her concern about ending counselling, and offer to continue with extra sessions until she is comfortable.
2. Recognise her concern about ending counselling, and share contacts of mental health professionals from whom she can seek further support.
3. Recognise her concern about ending counselling, and express your regret that you will not be able to continue with sessions.
4. Recognise her concern about ending counselling, and discuss how she can apply the skills she has learnt to future problems.

**Scenario B**

*Saurabh is a 17-year-old male from Mumbai who is studying in the 12th Standard. He lives with his uncle and aunt and their two sons (Saurabh’s cousins) who are aged 12 and 15 years. Saurabh’s parents live in a village, and they have recently sent him to the city for his education. It has been difficult for him to adjust to the new environment and he has not able to make new friends. Several of his classmates make fun of him and he is easily angered by this. He gets into fights at school and sometimes even outside of school. His teachers have spoken to Saurabh’s aunt and uncle to express concern about his anger outbursts. Saurabh has been warned by his aunt and uncle that he will be sent back to the village if he does not learn to control his temper and start getting along with his school peers.*

*Saurabh feels frustrated and has on occasion punched walls at home. He has experienced thoughts of hurting himself to feel better. His teacher has recommended that he attends counselling. He does not know how it will help, especially since he only gets angry when others are being unreasonable*

**Segment 1 instructions**: It is your first session with Saurabh and you are meeting him for the first time. Please answer questions 1-4.

**Saurabh says the following:**

*“My class teacher told me about counselling, but I don’t think talking about anything will help. The teachers and students in this school are terrible. My parents have sent me to live here with my uncle and aunt because our village doesn’t have good schools. These boys call me names and make fun of my family because we are from a village. I get so angry when they do that. I don’t think counselling can stop them from making fun of me and my family, can it?”*

Q 1. What might you say to begin the process of engaging Saurabh in counselling?

1. “It’s good to meet you. It is important that you understand how counselling can help you. If a person doesn’t believe it will be useful, then they are less likely to try.”
2. “It’s good to meet you. Let me begin by telling you how counselling works. It’s important to understand the benefits so you know that counselling is the right choice.”
3. “It’s good to meet you. I am sorry that students at school are making fun of you. Through counselling, we can think of ways to make them stop.”
4. “It’s good to meet you. It will be helpful for me to learn more about your problems. We can also discuss your concerns about counselling.”

Q 2. Saurabh tells you that he does not want anyone to know what he discusses with you in counselling sessions. How might you respond to Saurabh’s concern?

1. “Counselling sessions are confidential, but with limits. If your guardians or teachers ask me directly, I will have to tell them about our counselling sessions.”
2. “Counselling sessions are confidential, but with limits. If your safety seems to be at risk, then I would let you know before I inform anybody else.”
3. “Counselling sessions are confidential, but with limits. When your safety is at risk, I would have to inform your guardians. However, I will only break confidentiality if you give me permission.”
4. “Counselling sessions are confidential, but with limits. At times I may have to discuss your safety and progress with your guardian or teacher. Only if you agree with this can I continue counselling.”

Q 3. How might you introduce problem-solving counselling to Saurabh?

1. “In counselling, you will learn to recognize common problems related to anxiety and depression.”
2. “In counselling, you will learn strategies to communicate and adjust better with people around you.”
3. “In counselling, you will learn and practice how to manage problems through a series of steps.”
4. “In counselling, you will learn what causes your problems and then choose the best solutions.”

Q4. Which of the following would be an appropriate way to respond when Saurabh says counselling won’t help him?

1. Assure Saurabh that adolescents with similar problems have benefitted from counselling.
2. Assure Saurabh that he would be able to develop healthy relationships with people around him.
3. Assure Saurabh that he would be able to develop a calmer personality through counselling.
4. Assure Saurabh that as a counsellor you know how to improve his situation.

**Segment 2 instructions:** Your first session with Saurabh continues. Read the segment below and answer questions 5-8.

**Saurabh says the following:**

*“I don’t like feeling angry all the time and I don’t want to fight anymore. But I don’t know how it’s possible for this to change. I have tried talking to my teachers and my aunt, but nobody seems to care about me, they only care about themselves.*

*I am constantly worrying about all the things the other students have said about me and I am finding it difficult to sleep. I think it is best if I leave this school and go back to my village or if my father can get a job here so that at least I can be with my family.”*

Q 5. Which of the following would be an appropriate way to respond to Saurabh’s description of his problems?

1. “It seems like you are struggling a lot right now. It is common at your age to feel alone and angry with everyone.”
2. “It seems like you are struggling a lot right now. It’s common to feel angry and worried after moving away from family and being bullied at school .”
3. “It seems like you are struggling a lot right now. You might benefit from making new friends that are able to make you feel happy.”
4. “It seems like you are struggling a lot right now. Maybe we can start by focusing on effective ways to manage your anger.”

Q 6. Which of the following might be helpful to show that you have understood what Saurabh has said?

1. Ask him specific questions to ensure you have understood him clearly.
2. Ask him to summarize his main problems to ensure you have understood him clearly.
3. Provide a summary of the discussion to ensure you have understood him clearly.
4. Make notes of all his problems to ensure you have understood him clearly.

Q 7. How might you demonstrate warmth and understanding to Saurabh?

1. Tell him that he’s not alone and give him a small treat in his next session.
2. Tell him that it sounds like he is experiencing a lot of different feelings which can be overwhelming.
3. Tell him that you know what it feels like because you’ve been through a similar situation.
4. Tell him it’s okay to ask for help and that he can contact you any time he’s lonely.

Q 8. How might you begin working on the problem(s) that Saurabh is facing?

1. Encourage him to select the problem that you think is most distressing for him.
2. Encourage him to choose the problem that will be quickest to solve.
3. Encourage him to select the problem by listing and prioritizing them.
4. Encourage him to find solutions to all the problems that he has mentioned in the session.

**Segment 3 instructions**: It is your second session with Saurabh. Having agreed to work on anger outbursts as the target problem, you are now trying to facilitate option generation to help with managing the problem. Read the segment below and answer questions 9-10.

*“I am not sure what options are there to reduce my anger. I read the booklet you gave me, and it says that deep breathing or counting backwards could be helpful. [voice trembling] But what if these don’t work? [Eyes watering] Will it stop others from being mean? I don’t think these will work for me.”*

Q 9. Saurabh is visibly upset when describing his doubts about finding an effective solution. How might you adjust your body language while he is speaking?

1. Lean forward, and softly pat his arm to help him calm down.
2. Lean forward, and look away so that he doesn’t feel he is being watched.
3. Lean forward, and smile to show him your support.
4. Lean forward, and nod slightly to show you’ve heard what he’s saying.

Q 10. How might you introduce the process of generating options for solving Saurabh’s target problem? Select the statement that seems most appropriate.

1. “I will start making a list of options and you can add to the list.”
2. “If you start making a list of options, then we can review it together.”
3. “I can share a list of options that other adolescents have chosen. You can pick the ones you like."
4. “If you brainstorm options, I can tell you which ones to keep and which ones to discard.”

**Segment 4 instructions**: It is your third session with Saurabh. You are helping him to choose options for solving his target problem. Read the segment below and answer questions 11-13. Saurabh says the following:

*“I didn’t do my home practice of coming up with options because I didn’t see the point of it! These are all things that I must do but it’s not my fault that I get angry.”*

Q 11. Saurabh has not completed his home practice. Which of the following would be a suitable response?

1. Discuss the possibility of Saurabh asking a family member to remind him about doing his home practice.
2. Discuss obstacles to completing the home practice tasks and explore ways of overcoming these.
3. Discuss the downsides of not regularly completing home practice tasks.
4. Discuss whether Saurabh would prefer to practice all tasks in sessions rather than at home.

Q 12. How might you support Saurabh when it comes to choosing a preferred option for managing his anger?

1. Recommend an option that has worked for other adolescents and ask for his opinion on it.
2. Recommend an option that has a good evidence base from research.
3. Recommend that he gets inputs from his parents and then discusses it with you.
4. Recommend that he lists out the pros and cons of the options that have been generated.

Q 13. What is an important point to keep in mind while helping Saurabh create an action plan for his target problem?

1. The plan should be approved by his teachers and relatives so that they can help him with the potential solution.
2. The plan should be interesting so that he is motivated to implement the potential solution.
3. The plan should be specific about the situations in which he will try out the potential solution.
4. The plan should include a potential solution that has worked for adolescents in the past.

**Segment 5 instructions:**. Saurabh decides on an action plan that involves use of deep breathing to manage his feelings of anger. The following segment is from your fourth session with Saurabh, when you will be reviewing the implementation of the plan. Read the text below and answer questions 14-16.

**Saurabh says the following:**

*“I tried to take a few deep breaths when I felt angry, but I still ended up getting into a fight yesterday. It really doesn’t feel like the option is working. I am not sure if deep breathing is going to make me feel less angry.*

*I am tired of being so lonely all the time and living like this. I can’t go to the village and my parents can’t come here. I don’t want to live anymore.”*

Q 14. What would be an appropriate response to Saurabh’s statement that he doesn’t want to live any more?

1. Explain that he is young and has a lot to look forward to in his life.
2. Ask him if he has made any plans to act on his thoughts.
3. Refocus on the agenda to avoid encouraging him to end his life.
4. Enquire about the purpose and benefits of ending his life.

Q 15. Which of the following is NOT a step in reviewing the implementation of the action plan with Saurabh?

1. Praise the efforts Saurabh made in implementing the plan.
2. Discuss the steps Saurabh took in implementing the plan.
3. Provide feedback on where Saurabh went wrong in implementing the plan.
4. Help Saurabh think of ways to overcome the challenges faced in implementing the plan.

Q 16. Which of the following would NOT be an appropriate way to help Saurabh overcome the challenges faced in implementing his plan?

1. Suggest that he can work with you to modify the parts of his plan that were not helpful.
2. Suggest that he can go back to the list of options generated earlier and choose a new option to include in his plan.
3. Suggest that he continues practicing deep breathing as this will take time to show effect.
4. Suggest that attempting to manage his anger seems challenging currently, and it may be helpful to focus on a different problem for now.
